# 2,2-Dimethyl-5-(2,3,4-trimeth­oxy­benzyl­idene)-1,3-dioxane-4,6-dione

**DOI:** 10.1107/S1600536811026201

**Published:** 2011-07-06

**Authors:** Wu-Lan Zeng

**Affiliations:** a MicroScale Science Institute, Department of Chemistry and Chemical Engineering, Weifang University, Weifang 261061, People’s Republic of China

## Abstract

The title compound, C_16_H_18_O_7_, was prepared by the reaction of 2,2-dimethyl-1,3-dioxane-4,6-dione and 2,3,4-trimeth­oxy­benzaldehyde. The 1,3-dioxane ring is in a slightly distorted boat conformation. The crystal structure is stabilized by weak inter­molecular C—H⋯O hydrogen bonds.

## Related literature

For related structures, see: Zeng (2011*a* ▶,*b* ▶).
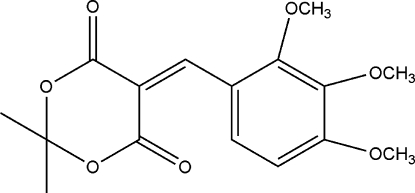


## Experimental

### 

#### Crystal data

C_16_H_18_O_7_
*M* *_r_* = 322.30
Monoclinic, 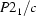

*a* = 12.722 (3) Å
*b* = 9.2669 (19) Å
*c* = 13.537 (3) Å
β = 98.83 (3)°
*V* = 1577.0 (5) Å^3^
*Z* = 4
Mo *K*α radiation
μ = 0.11 mm^−1^
*T* = 293 K
0.22 × 0.18 × 0.16 mm

#### Data collection

Bruker SMART CCD diffractometer
14870 measured reflections
3613 independent reflections
3073 reflections with *I* > 2σ(*I*)
*R*_int_ = 0.033

#### Refinement

*R*[*F* ^2^ > 2σ(*F* ^2^)] = 0.043
*wR*(*F* ^2^) = 0.131
*S* = 1.03
3613 reflections
208 parameters
H-atom parameters constrained
Δρ_max_ = 0.33 e Å^−3^
Δρ_min_ = −0.25 e Å^−3^

### 

Data collection: *SMART* (Bruker, 1997 ▶); cell refinement: *SAINT* (Bruker, 1997 ▶); data reduction: *SAINT*; program(s) used to solve structure: *SHELXS97* (Sheldrick, 2008 ▶); program(s) used to refine structure: *SHELXL97* (Sheldrick, 2008 ▶); molecular graphics: *SHELXTL* (Sheldrick, 2008 ▶); software used to prepare material for publication: *SHELXTL*.

## Supplementary Material

Crystal structure: contains datablock(s) global, I. DOI: 10.1107/S1600536811026201/lh5270sup1.cif

Structure factors: contains datablock(s) I. DOI: 10.1107/S1600536811026201/lh5270Isup2.hkl

Supplementary material file. DOI: 10.1107/S1600536811026201/lh5270Isup3.cml

Additional supplementary materials:  crystallographic information; 3D view; checkCIF report

## Figures and Tables

**Table 1:** Hydrogen-bond geometry (Å, °)

*D*—H⋯*A*	*D*—H	H⋯*A*	*D*⋯*A*	*D*—H⋯*A*
C3—H3*A*⋯O4^i^	0.96	2.46	3.392 (2)	163
C16—H16*B*⋯O5^ii^	0.96	2.58	3.397 (2)	143

Symmetry codes: (i) 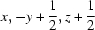; (ii) 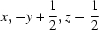.

**Fig. 1.**
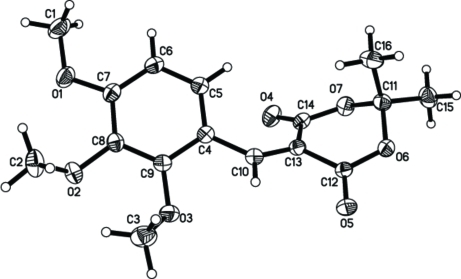
The molecular structure of (I), drawn with 30% probability ellipsoids and spheres of arbritrary size for the H atoms.

## supplementary crystallographic information

### Comment

In previous papers the crystal structures of 2,2-Dimethyl-5-[(5-methylfuran-2-yl)methylidene]-1,3- dioxane-4,6-dione and (5-(3,4-Dimethylbenzylidene)-2,2- dimethyl-1,3-dioxane-4,6-dione are reported (Zeng. 2011a,b). As part of the search for new Meldrum's acids, the molecular structure of title compound (I) has been synthesized and its crystal structure is reported herein. The crystal structure of the title compound is shown in Fig. 1. The 1,3-dioxane ring exhibits a slightly distorted boat conformation. The crystal structure is stabilized by weak intermolecular C—H···O hydrogen bonds (Table 1).

### Experimental

A mixture of malonic acid (6.24 g, 0.06 mol) and acetic anhydride(9 ml) in strong sulfuric acid (0.25 ml) was stirred with water at 303K. After dissolving, propan-2-one (3.48 g, 0.06 mol) was added dropwise into the solution for 1 h. The reaction was allowed to proceed for 2 h. The mixture was cooled and filtered, and then an ethanol solution of 2,3,4-trimethoxybenzaldehyde (11.76g,0.06 mol) was added. The solution was then filtered and concentrated. Single crystals were obtained by evaporation of an petroleum ether-ethylacetate (3:1 *v*/*v*) solution of (I) at room temperature over a period of several days.

### Refinement

The H atoms were placed in calculated positions (C—H = 0.93–0.96 Å), and refined as riding with U_iso_(H) = 1.2U_eq_(C) or 1.5U_eq_(methyl C).

### Crystal data

**Table:**

C_16_H_18_O_7_	*F*(000) = 680
*M**_r_* = 322.30	*D*_x_ = 1.358 Mg m^−^^3^
Monoclinic, *P*2_1_/*c*	Mo *K*α radiation, λ = 0.71073 Å
Hall symbol: -P 2ybc	Cell parameters from 3073 reflections
*a* = 12.722 (3) Å	θ = 3.0–27.5°
*b* = 9.2669 (19) Å	µ = 0.11 mm^−^^1^
*c* = 13.537 (3) Å	*T* = 293 K
β = 98.83 (3)°	Block, yellow
*V* = 1577.0 (5) Å^3^	0.22 × 0.18 × 0.16 mm
*Z* = 4	

### Data collection

**Table:**

Bruker SMART CCD diffractometer	3073 reflections with *I* > 2σ(*I*)
Radiation source: fine-focus sealed tube	*R*_int_ = 0.033
graphite	θ_max_ = 27.5°, θ_min_ = 3.0°
φ and ω scans	*h* = −16→16
14870 measured reflections	*k* = −12→10
3613 independent reflections	*l* = −17→17

### Refinement

**Table:**

Refinement on *F*^2^	Primary atom site location: structure-invariant direct methods
Least-squares matrix: full	Secondary atom site location: difference Fourier map
*R*[*F*^2^ > 2σ(*F*^2^)] = 0.043	Hydrogen site location: inferred from neighbouring sites
*wR*(*F*^2^) = 0.131	H-atom parameters constrained
*S* = 1.03	*w* = 1/[σ^2^(*F*_o_^2^) + (0.0828*P*)^2^ + 0.1973*P*] where *P* = (*F*_o_^2^ + 2*F*_c_^2^)/3
3613 reflections	(Δ/σ)_max_ < 0.001
208 parameters	Δρ_max_ = 0.33 e Å^−^^3^
0 restraints	Δρ_min_ = −0.25 e Å^−^^3^

### Special details

**Table:**

Geometry. All e.s.d.'s (except the e.s.d. in the dihedral angle between two l.s. planes) are estimated using the full covariance matrix. The cell e.s.d.'s are taken into account individually in the estimation of e.s.d.'s in distances, angles and torsion angles; correlations between e.s.d.'s in cell parameters are only used when they are defined by crystal symmetry. An approximate (isotropic) treatment of cell e.s.d.'s is used for estimating e.s.d.'s involving l.s. planes.
Refinement. Refinement of *F*^2^ against ALL reflections. The weighted *R*-factor *wR* and goodness of fit *S* are based on *F*^2^, conventional *R*-factors *R* are based on *F*, with *F* set to zero for negative *F*^2^. The threshold expression of *F*^2^ > σ(*F*^2^) is used only for calculating *R*-factors(gt) *etc*. and is not relevant to the choice of reflections for refinement. *R*-factors based on *F*^2^ are statistically about twice as large as those based on *F*, and *R*- factors based on ALL data will be even larger.

### Fractional atomic coordinates and isotropic or equivalent isotropic displacement parameters (Å^2^)

**Table:**

	*x*	*y*	*z*	*U*_iso_*/*U*_eq_	
O2	0.52394 (8)	0.12332 (10)	0.25642 (7)	0.0477 (2)	
O3	0.30226 (7)	0.11922 (11)	0.24083 (6)	0.0475 (2)	
O7	0.01954 (7)	0.41504 (10)	−0.11404 (7)	0.0455 (2)	
O5	−0.04628 (7)	0.11016 (11)	0.07142 (6)	0.0470 (2)	
O6	−0.09078 (7)	0.23104 (10)	−0.06837 (6)	0.0423 (2)	
O4	0.17489 (8)	0.46436 (11)	−0.02499 (9)	0.0549 (3)	
O1	0.61501 (7)	0.18353 (12)	0.09474 (8)	0.0535 (3)	
C14	0.10299 (10)	0.38024 (13)	−0.04308 (9)	0.0390 (3)	
C9	0.35150 (10)	0.14559 (12)	0.15946 (9)	0.0373 (3)	
C13	0.09149 (9)	0.24390 (13)	0.01095 (8)	0.0361 (3)	
C12	−0.01832 (9)	0.18810 (13)	0.00905 (8)	0.0361 (3)	
C8	0.46155 (10)	0.14555 (13)	0.16543 (9)	0.0387 (3)	
C4	0.28552 (10)	0.18482 (13)	0.07028 (9)	0.0390 (3)	
C7	0.50721 (10)	0.18263 (13)	0.08077 (10)	0.0413 (3)	
C6	0.44249 (11)	0.21837 (16)	−0.00816 (10)	0.0467 (3)	
H6A	0.4726	0.2408	−0.0647	0.056*	
C5	0.33352 (11)	0.22040 (15)	−0.01229 (10)	0.0455 (3)	
H5A	0.2910	0.2462	−0.0718	0.055*	
C10	0.17081 (10)	0.17055 (14)	0.06602 (9)	0.0395 (3)	
H10A	0.1493	0.1002	0.1076	0.047*	
C11	−0.05435 (10)	0.30305 (14)	−0.15114 (9)	0.0414 (3)	
C15	−0.15002 (13)	0.37887 (18)	−0.20694 (12)	0.0561 (4)	
H15A	−0.1781	0.4445	−0.1628	0.084*	
H15B	−0.2033	0.3090	−0.2316	0.084*	
H15C	−0.1296	0.4315	−0.2621	0.084*	
C3	0.32531 (14)	−0.01527 (18)	0.29164 (13)	0.0626 (4)	
H3A	0.2861	−0.0216	0.3467	0.094*	
H3B	0.4001	−0.0210	0.3162	0.094*	
H3C	0.3052	−0.0934	0.2461	0.094*	
C2	0.58511 (12)	−0.00798 (17)	0.26325 (14)	0.0616 (4)	
H2A	0.6262	−0.0156	0.3287	0.092*	
H2B	0.6320	−0.0068	0.2141	0.092*	
H2C	0.5379	−0.0891	0.2515	0.092*	
C16	−0.00279 (14)	0.19646 (17)	−0.21299 (10)	0.0563 (4)	
H16A	0.0574	0.1530	−0.1724	0.084*	
H16B	0.0203	0.2456	−0.2683	0.084*	
H16C	−0.0532	0.1230	−0.2377	0.084*	
C1	0.66751 (13)	0.2140 (2)	0.01138 (14)	0.0695 (5)	
H1A	0.7431	0.2112	0.0321	0.104*	
H1B	0.6471	0.3082	−0.0143	0.104*	
H1C	0.6476	0.1432	−0.0399	0.104*	

### Atomic displacement parameters (Å^2^)

**Table:**

	*U*^11^	*U*^22^	*U*^33^	*U*^12^	*U*^13^	*U*^23^
O2	0.0458 (5)	0.0472 (5)	0.0461 (5)	−0.0004 (4)	−0.0054 (4)	0.0007 (4)
O3	0.0486 (5)	0.0564 (6)	0.0394 (5)	0.0052 (4)	0.0130 (4)	0.0080 (4)
O7	0.0446 (5)	0.0398 (5)	0.0518 (5)	0.0001 (4)	0.0062 (4)	0.0080 (4)
O5	0.0473 (5)	0.0564 (5)	0.0399 (5)	−0.0060 (4)	0.0141 (4)	0.0081 (4)
O6	0.0350 (4)	0.0527 (5)	0.0392 (4)	−0.0028 (4)	0.0058 (3)	0.0051 (4)
O4	0.0456 (5)	0.0437 (5)	0.0765 (7)	−0.0090 (4)	0.0134 (5)	−0.0020 (5)
O1	0.0344 (5)	0.0634 (6)	0.0636 (6)	−0.0038 (4)	0.0109 (4)	−0.0018 (5)
C14	0.0361 (6)	0.0377 (6)	0.0455 (6)	0.0008 (5)	0.0134 (5)	−0.0020 (5)
C9	0.0402 (6)	0.0364 (6)	0.0360 (6)	−0.0007 (5)	0.0082 (5)	−0.0009 (4)
C13	0.0350 (6)	0.0407 (6)	0.0336 (5)	−0.0019 (4)	0.0082 (4)	−0.0017 (4)
C12	0.0356 (6)	0.0412 (6)	0.0329 (5)	−0.0005 (4)	0.0096 (4)	−0.0022 (4)
C8	0.0385 (6)	0.0352 (6)	0.0408 (6)	−0.0010 (5)	0.0012 (5)	−0.0018 (4)
C4	0.0358 (6)	0.0426 (6)	0.0389 (6)	0.0011 (5)	0.0072 (5)	0.0003 (5)
C7	0.0359 (6)	0.0390 (6)	0.0499 (7)	−0.0017 (5)	0.0097 (5)	−0.0046 (5)
C6	0.0438 (7)	0.0554 (7)	0.0435 (7)	0.0023 (6)	0.0149 (5)	0.0022 (6)
C5	0.0421 (7)	0.0567 (8)	0.0381 (6)	0.0051 (6)	0.0072 (5)	0.0042 (5)
C10	0.0390 (6)	0.0454 (6)	0.0349 (5)	−0.0004 (5)	0.0079 (5)	0.0008 (5)
C11	0.0442 (7)	0.0437 (6)	0.0364 (6)	−0.0003 (5)	0.0063 (5)	0.0044 (5)
C15	0.0520 (8)	0.0605 (9)	0.0524 (8)	0.0052 (6)	−0.0034 (6)	0.0089 (6)
C3	0.0726 (10)	0.0571 (9)	0.0623 (9)	−0.0038 (7)	0.0240 (8)	0.0149 (7)
C2	0.0482 (8)	0.0536 (8)	0.0775 (10)	0.0039 (6)	−0.0074 (7)	0.0128 (7)
C16	0.0732 (10)	0.0582 (8)	0.0390 (6)	0.0102 (7)	0.0131 (6)	0.0002 (6)
C1	0.0452 (8)	0.0925 (13)	0.0762 (11)	−0.0078 (8)	0.0267 (8)	−0.0085 (9)

### Geometric parameters (Å, °)

**Table:**

O2—C8	1.3745 (15)	C6—C5	1.3789 (19)
O2—C2	1.4396 (18)	C6—H6A	0.9300
O3—C9	1.3702 (14)	C5—H5A	0.9300
O3—C3	1.4319 (18)	C10—H10A	0.9300
O7—C14	1.3567 (16)	C11—C15	1.5044 (19)
O7—C11	1.4379 (16)	C11—C16	1.5085 (19)
O5—C12	1.2056 (14)	C15—H15A	0.9600
O6—C12	1.3453 (15)	C15—H15B	0.9600
O6—C11	1.4406 (14)	C15—H15C	0.9600
O4—C14	1.1981 (15)	C3—H3A	0.9600
O1—C7	1.3553 (15)	C3—H3B	0.9600
O1—C1	1.4251 (19)	C3—H3C	0.9600
C14—C13	1.4783 (17)	C2—H2A	0.9600
C9—C8	1.3898 (17)	C2—H2B	0.9600
C9—C4	1.4082 (17)	C2—H2C	0.9600
C13—C10	1.3436 (17)	C16—H16A	0.9600
C13—C12	1.4861 (16)	C16—H16B	0.9600
C8—C7	1.4048 (18)	C16—H16C	0.9600
C4—C5	1.3931 (17)	C1—H1A	0.9600
C4—C10	1.4576 (17)	C1—H1B	0.9600
C7—C6	1.3901 (19)	C1—H1C	0.9600
			
C8—O2—C2	114.60 (11)	O7—C11—C15	105.86 (11)
C9—O3—C3	117.14 (11)	O6—C11—C15	105.95 (11)
C14—O7—C11	118.31 (10)	O7—C11—C16	110.48 (11)
C12—O6—C11	118.63 (9)	O6—C11—C16	110.34 (11)
C7—O1—C1	118.55 (12)	C15—C11—C16	114.48 (12)
O4—C14—O7	118.68 (12)	C11—C15—H15A	109.5
O4—C14—C13	125.83 (12)	C11—C15—H15B	109.5
O7—C14—C13	115.29 (10)	H15A—C15—H15B	109.5
O3—C9—C8	122.28 (11)	C11—C15—H15C	109.5
O3—C9—C4	116.70 (11)	H15A—C15—H15C	109.5
C8—C9—C4	120.82 (11)	H15B—C15—H15C	109.5
C10—C13—C14	125.73 (11)	O3—C3—H3A	109.5
C10—C13—C12	117.23 (11)	O3—C3—H3B	109.5
C14—C13—C12	116.94 (10)	H3A—C3—H3B	109.5
O5—C12—O6	118.84 (11)	O3—C3—H3C	109.5
O5—C12—C13	125.03 (11)	H3A—C3—H3C	109.5
O6—C12—C13	116.13 (10)	H3B—C3—H3C	109.5
O2—C8—C9	119.34 (11)	O2—C2—H2A	109.5
O2—C8—C7	120.90 (11)	O2—C2—H2B	109.5
C9—C8—C7	119.44 (11)	H2A—C2—H2B	109.5
C5—C4—C9	118.16 (11)	O2—C2—H2C	109.5
C5—C4—C10	123.32 (12)	H2A—C2—H2C	109.5
C9—C4—C10	118.16 (11)	H2B—C2—H2C	109.5
O1—C7—C6	124.85 (12)	C11—C16—H16A	109.5
O1—C7—C8	115.09 (12)	C11—C16—H16B	109.5
C6—C7—C8	120.04 (11)	H16A—C16—H16B	109.5
C5—C6—C7	119.77 (12)	C11—C16—H16C	109.5
C5—C6—H6A	120.1	H16A—C16—H16C	109.5
C7—C6—H6A	120.1	H16B—C16—H16C	109.5
C6—C5—C4	121.74 (12)	O1—C1—H1A	109.5
C6—C5—H5A	119.1	O1—C1—H1B	109.5
C4—C5—H5A	119.1	H1A—C1—H1B	109.5
C13—C10—C4	129.65 (11)	O1—C1—H1C	109.5
C13—C10—H10A	115.2	H1A—C1—H1C	109.5
C4—C10—H10A	115.2	H1B—C1—H1C	109.5
O7—C11—O6	109.50 (10)		

### Hydrogen-bond geometry (Å, °)

**Table:**

*D*—H···*A*	*D*—H	H···*A*	*D*···*A*	*D*—H···*A*
C3—H3A···O4^i^	0.96	2.46	3.392 (2)	163
C16—H16B···O5^ii^	0.96	2.58	3.397 (2)	143

Symmetry codes: (i) *x*, −*y*+1/2, *z*+1/2; (ii) *x*, −*y*+1/2, *z*−1/2.
